# Synergistic anticancer efficacy of optimized curcumin-piperine loaded magnetic nanoparticles for the treatment of colorectal cancer

**DOI:** 10.37349/etat.2025.1002340

**Published:** 2025-10-20

**Authors:** Ritika Puri, Vimal Arora

**Affiliations:** University of Valladolid, Spain; University Institute of Pharma Sciences, Chandigarh University, Gharuan 140413, Punjab, India

**Keywords:** magnetic nanoparticles, HCT-116 colon cell line, synergistic effect, curcumin, piperine

## Abstract

**Aim:**

The current study uses the depicted approach to synthesize curcumin-piperine loaded Poloxamer F-68 coated magnetic nanoparticles (CUR-PIP-F68-Fe_3_O_4_ NPs) to achieve a synergistic anti-cancer impact on an in vitro HCT-116 colon cancer cell. Integrating magnetic nanoparticle technology with phytoconstituents enhances the potential for targeted drug delivery with minimal systemic toxicity and facilitates therapeutic outcomes.

**Methods:**

A Box-Behnken design was employed to optimize the CUR-PIP-F68-Fe_3_O_4 _NPs prepared by the co-precipitation method. Optimized formulation was evaluated for morphological characteristics, elemental composition, and magnetic properties. An in vitro cytotoxicity assay was conducted to observe the % viability of cells and to further calculate the IC50. Cellular uptake studies were investigated using confocal microscopy.

**Results:**

Results showed that the optimised nanoparticles possessed a particle size of 158.7 ± 0.057 nm, zeta potential of –30.3 ± 0.1 mV, and encapsulation efficiency of 98.85 ± 0.066%. Analysis by vibrational sample magnetometer revealed that magnetic saturation was 75.6 emu/g and 50.7 emu/g for bare Fe_3_O_4 _nanoparticles and drug-loaded magnetic nanoparticles, respectively. Scanning electron microscopy (SEM) depicted the morphological characteristics; elemental composition of synthesized magnetic nanoparticles was confirmed by energy dispersive X-ray (EDX) analysis by illustrating the presence of C (13.50 ± 0.30%), Fe (78.81 ± 1.23%), and O (7.69 ± 0.29%). The MTT assay and cellular uptake studies unveiled that CUR-PIP-loaded magnetic nanoparticles possess a synergistic cytotoxic effect and the highest drug uptake against the HCT-116 colon cell line.

**Conclusions:**

The combination approach of curcumin-piperine magnetic nanoparticles to HCT-116 cells enhanced the anticancer efficacy of the curcumin and further demonstrated the potential of this approach to conduct in vivo studies.

## Introduction

Cancer is a disease of unregulated cell growth that can affect any body part and develop beyond its usual limits, invading adjoining tissues and spreading to other organs [1, 2]. As per the World Health Organization report, 19.9 million new cases and 9.7 million deaths were reported in 2022 due to various types of cancer, and annual new cases are expected to upsurge to 27.6 million globally by 2030 [3]. According to GLOBOCAN 2022 data, lung cancer is the leading cause of cancer deaths, reportedly 1.8 million (18.7%), followed by colorectal accounting for roughly 0.9 million deaths (9.3%), liver cancer (7.8%), breast cancer (6.8%), stomach cancer (6.5%), and pancreatic cancer (4.8%) [4]. The number of colorectal cases and death rate at the global level reflects the burden of cancer incidence and mortality associated with it, which provokes and demands thinking upon the risk factors associated with and/or initiating the cancer, and a promising solution for their management.

Several drugs are available for colorectal cancer, but for known and unknown reasons, most of these drugs are ineffective, expensive, and have safety concerns. One of the prime reasons for their failure is the single target for action, such as Coxibs (Cyclooxygenase or COX-2), Erbuitux (Epidermal Growth Factor Receptor), and Avastin (Vascular Endothelial Growth Factor or VEGF) [5]. Another reason for the failure of existing treatments is the inability of commercially available chemotherapeutic agents or existing therapies to kill cancer cells. This is often due to challenges associated with the systemic route, leading to variations in absorption, distribution, metabolism, as well as a failure to deliver drugs to target tissues. Furthermore, heterogeneous drug distribution within the tumour region and systemic side effects such as multi-drug resistance, bone marrow suppression, and hair loss also limit their efficacy [6–8].

To overcome the above limitations, this proposed research work is aligned with an approach of blending the traditional system of medicine (phytoconstituent) and novel technology (magnetic nanoparticles) for effective and affordable treatment of cancer.

Curcumin, an active component of *Curcuma longa*, is one of the eminent species mostly cultivated in warm climates around the globe [9], and its rhizomes are the most widely used part [10], containing bioactive non-volatile and volatile compounds [11]. Curcumin demonstrates a complex mechanism of action to fight cancer cells. Curcumin helps in regulating cell proliferation and causes apoptosis by inhibiting the NF-κB and Akt/mTOR pathways, or modifying the MAPK pathway, or by changing Bcl-2 family proteins and activating caspases [12]. Moreover, curcumin suppresses pro-angiogenic factors such as VEGF and bFGF and impairs the functions of endothelial cells to prevent angiogenesis. Furthermore, curcumin modifies histone enzymes, influences DNA methylation, and changes microRNA expression to impact epigenetic regulation [13, 14].

Despite a proven number of therapeutic properties, Curcumin has many obstacles that prevent it from being used effectively in clinical settings. Its low bioavailability is one of the most significant problems. Due to its low water solubility (0.008 mg/mL), curcumin is poorly absorbed in the digestive system, restricting its potential therapeutic use. Curcumin’s fast metabolism in the liver and intestinal walls results in a short plasma half-life and decreased availability in the bloodstream. In the liver, curcumin is rapidly conjugated to glucuronides and sulfates, forming more water-soluble conjugates that facilitate their excretion via urine [15].

Additionally, curcumin is metabolized into several products: dihydrocurcumin, a reduced form of curcumin, which has potentially different biological activities; tetrahydrocurcumin, another reduced form that is more stable and also found in the urine; and hexahydrocurcumin, which features a fully saturated ring system and may exhibit distinct biological activities from curcumin. These metabolic products, along with curcumin glucuronides and curcumin sulfates, contribute to the overall low bioavailability and reduced therapeutic effectiveness of curcumin. Another challenge is the instability of curcumin, which is prone to degradation under physiological conditions, particularly at neutral and alkaline pH, further complicating its effectiveness. Lower absorption and rapid excretion from the body, and hence limited bioavailability, necessitate large doses to maintain stable therapeutic levels, which can be impractical [16].

### Combination approach with piperine

Piperine, a bioactive compound with the formula C_17_H_19_NO_3_ present in black pepper (*Piper nigrum*), has been reported to lower cancer incidence in rat lung cancer models [17]. It has been extensively documented that piperine inhibits the proliferation of colon cancer cell lines by inducing apoptosis and G1 arrest in the cell cycle [18]. Piperine also significantly enhances curcumin’s bioavailability by enhancing intestinal absorption and inhibiting certain enzymes that participate in drug metabolism (e.g., cytochrome P450) [19, 20]. Many studies have reported that the co-delivery of piperine with curcumin can increase curcumin’s bioavailability by up to 2000%. By combining curcumin and piperine, formulations can achieve therapeutic concentrations of curcumin that would otherwise not be possible with curcumin alone. This makes the combination more effective in treating conditions such as various cancers, arthritis, and cardiovascular disorders [21, 22].

Magnetic nanoparticles (MNPs) are a type of nanomaterial composed of metal substances like iron, nickel, cobalt, chromium, and manganese and their derivatives. MNPs have the significant advantage of being magnetically modulated with an external magnetic field to target certain organs or tissues [23]. Primarily, MNPs are comprised of an inner core shell made up of magnetite (Fe_3_O_4_) or maghemite Fe_2_O_3_ and a coating material (polymers). Their ability to concentrate curcumin at tumor locations while reducing off-target effects makes MNPs especially helpful in precision therapy [24]. Moreover, imaging applications can make use of their magnetic characteristics. In a previous study, curcumin MNPs showed a preferential uptake in MDA-MB-231 cells in a concentration-dependent manner and demonstrated accumulation throughout the cell, which indicates that particles are internalized through endocytosis [23, 24]. Another recent study published the synergistic effect of curcumin-piperine on MCF-7 breast cancer cells and showed significant cytotoxicity as compared to the individual drug [25].

However, there is no specific research available on the optimization of MNPs and uptake studies against the HCT-116 human colorectal carcinoma cell line. The present study synthesizes a hybrid system that provides a novel treatment to synergistic cancer treatment by delivering two natural compounds via a therapeutically translatable and magnetically responsive nanocarrier, providing new evidence on the effectiveness of the curcumin-piperine combination delivered. The combined effect of the nanosystem was investigated using cell viability and cellular uptake analysis to give more evidence on the interaction of two active phytoconstituents with HCT-116 cancer cells.

## Materials and methods

### Materials

Piperine was procured from Thermo Scientific Chemicals (Cat. no. A13510). Curcumin (99%), ferric chloride, and ferrous sulphate were bought from Central Drug House (CDH) Pvt. Ltd., Delhi (Cat. no. 020031). Poloxamer-F68 (Pluronic^®^F68) from HiMedia Laboratories, Cat. no. TC222, Mumbai, India. Every other chemical utilized in the investigation was acquired from nearby suppliers and is of analytical grade.

### Methods

#### Synthesis of MNPs

**Figure 1:**
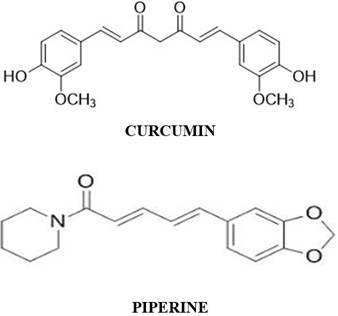
Chemical structure of curcumin and piperine.

Ferrous sulfate tetrahydrate and ferric chloride hexahydrate were prepared in a molar ratio of 1:2 (by taking 4.63 g of Ferrous sulfate tetrahydrate and 9.00 g of ferric chloride hexahydrate in distilled water) to prepare the Fe_3_O_4_ nanoparticles by the co-precipitation method as illustrated in Figure 1. Prepared mixture was continuously sonicated using a probe sonicator (Pro 650, LabMan) at 65°C, followed by dropwise addition of ammonium hydroxide solution till the color changed from brown to black and pH reached 12 [23]. Stirring was continued for 30 minutes and then continuously purged with N_2_ for another 10 minutes. The precipitates were then allowed to settle for 12 h at room temperature, followed by centrifugation and multiple washes with triple-distilled water. The collected precipitate was then dried at 60°C in a vacuum oven for 1 h. The final product, Fe_3_O_4_-NP, was obtained as a black powder.

Chemical reaction is expressed as:

4FeSO_4_ + 2FeCl_3_ + 10NH_4_OH → Fe_3_O_4_ + 8NH_4_Cl + 10H_2_O

#### Coating of MNPs

For coating of iron oxide nanoparticles, a 1% w/v solution of poloxamer F-68 polymer was prepared by dissolving in distilled water at a temperature of 60 ± 0.5°C, and approximately 200 mg of iron oxide nanoparticles were added and mixed using a magnetic stirrer for 12 h. Coated particles were separated by the use of a permanent magnet and dried in a vacuum at 60 ± 0.5°C.

#### Formulation of drug-loaded MNPs

Curcumin-piperine drug loading for the MNPs based on the Box-Behnken statistical design F1 to F17 was carried out by dispersing 100 mg of poloxamer F-68-coated MNPs in water and stirring with a magnetic stirrer (REMI Lab World, Mumbai) at 1500 rpm. In another beaker, 20 mg of curcumin and 10 mg of piperine were dissolved in methanol and added to the coated-MNPs solution under continuous magnetic stirring for 24 h to facilitate drug uptake. After 24 h, MNPs were collected using the magnet, washed with distilled water, and dried using a vacuum oven (RVO-50, REMI Lab World) at 60 ± 0.5°C.

#### Optimization of curcumin piperine-loaded MNPs using Box-Behnken statistical design

Stat-Ease (Stat-Ease Inc., Minneapolis, USA) Design Expert^®^ 13 software was used to apply the Box-Behnken design to optimise the Fe_3_O_4_ nanoparticles, mainly focusing on three critical factors: concentrations of magnetite (A), coating solution (B), and sonication time (C). The critical responses chosen for this study were particle size (R1), zeta potential (R2), and encapsulation efficiency (R3). The maximum and minimum level value given to each independent variable is represented in Table 1.

**Table 1:** Variables used for the formation and optimization of CUR-PIP-loaded MNPs by Box-Behnken design.

**Factors**	**Levels**		
**Independent variables**	**Unit**	**Low**	**High**
**A: Concentration of magnetite**	%w/v	0.1	0.4
**B: Concentration of coating solution**	%w/v	0.5	1.5
**C: Sonication time**	minutes	30	60
**Dependent variables**	**Goals**		
**R1: Particle size**	Minimize		
**R2: Zeta potential**	Maximise		
**R3: Encapsulation efficiency**	Maximize		

### Characterization

#### Particle size and polydispersity index

Particle size analysis and polydispersity index (PDI) determination of the synthesized iron oxide nanoparticles, MNPs, after performing coating and formulation of curcumin-piperine loaded iron oxide nanoparticles, were conducted utilizing a particle size analyser (Nano ZS, Malvern Instruments, UK) by dispersing prepared MNPs in distilled water [26].

#### Zeta potential determination

The zeta potential of bare MNPs, MNPs with polymer coating, and CUR-PIP-F68-Fe_3_O_4_ nanoparticles was measured using a zeta sizer (Anton Parr, Litesizer DLS 500). The sample was sonicated for 5 min [27], and the dispersion was prepared using water (10 mL).

#### Scanning electron microscopy-energy dispersive X-ray (SEM-EDX) analysis

SEM-EDX analysis of the synthesized bare MNPs and drug-loaded iron oxide nanoparticles was conducted using an SEM (JSM 6100, Jeol) to investigate their morphology and elemental composition. For this analysis, a small amount of the nanoparticle sample was dispersed onto a conductive substrate, and a gold coating was performed to prevent charging under the electron beam [28].

#### Transmission electron microscopy (TEM)

The morphology of the formed nanoparticles was examined with a transmission electron microscope (Technai, FEI Co., The Netherlands). For this, one mL of nanodispersion was diluted 100 times and one drop of diluted dispersion was placed on a copper grid alongside 2% phosphotungstic acid, which served as a negative stain. The constructed grid was dried and examined under a microscope at an appropriate magnification to obtain microphotographs [29].

#### Vibrating simulating magnetometry

During vibrating simulating magnetometer (VSM) measurement, the magnetization of the sample aligns with the external magnetic field. To study the magnetic characteristics of the synthesized MNPs, the experiment was performed on a vibrating sample magnetometer (7410 series, Lake Shore Cryotronics, United States) at room temperature up to a magnetic field of 2 T.

#### Encapsulation efficiency

The encapsulation efficiency (EE) of the formulated CUR-PIP-F68-Fe_3_O_4_ was determined using an indirect method by estimating the total amount of free curcumin and piperine in the supernatant [30]. For this purpose, 10 mg of the drug-loaded MNPs was added to 10 mL of distilled water. The mixture was then centrifuged using a centrifuge machine at 5,000 rpm for 30 min. After centrifugation, the supernatant containing free drug was separated from the pellet. The concentration of free curcumin or piperine in the supernatant was determined by HPLC (Agilent 1220 Infinity LC equipped with UV detector) at λ_max_ of 376 nm (isosbestic point of curcumin and piperine) after appropriately diluting the sample. The percentage encapsulation efficiency (%EE) of the MNPs was calculated using the following equation:

$$\%EE\;\left(curcumin\;or\;piperine\right)=\frac{Total\;Drug\;Concentration-Free\;Drug\;Concentration}{Total\;Drug\;Concentration}\times 100$$

#### In vitro cytotoxicity assay

To conduct this experiment, the HCT-116 cell line was procured from NCCS Pune. 10,000 cells were grown for 24 h in a plate containing 96 wells, maintaining the temperature at 37 ± 0.5°C with 5% CO_2_ in Dulbecco’s Modified Eagle media- AT149 containing 10% fetal bovine serum (HIMEDIA-RM 10432) and 1% antibiotic solution (Sigma-Aldrich P0781) [31]. The subsequent day, cells were exposed to various concentrations of curcumin-piperine free drug, curcumin MNPs, piperine MNPs, and their combinations. MTT (5 mg/mL) was incorporated into the cell culture after it had been incubated for 24 h, followed by incubation for an additional 2 h in a CO_2_ incubator with an air jacket (Heal Force-HF90, Shanghai, China). Following incubation, the culture medium containing MTT was carefully removed without disturbing the formazan crystals, and the cell layer matrix was dissolved in 100 µL Dimethyl Sulfoxide (SRL- Cat no.- 67685), and read in an ELISA plate reader (iMark, Biorad, USA) at 540 nm. The half-maximal inhibitory concentration (IC50) was estimated using GraphPad Prism-6. Pictures were taken by an AmScope digital camera (Aptima CMOS). Mean ± standard error of the mean (SEM) was used to present the data.

$$\%\;Viable\;cells=(\frac{A{bsorbance}_{test}}{{Absorbance}_{Control}})\times 100$$

#### Cellular uptake of compounds in the cell line—HCT-116

Ultraclean coverslips were placed in 6-well plates, followed by the addition of 200 µL 3-aminopropyltriethoxysilane (APTES) (APTES is a silane coupling agent that is commonly used to modify the surface of sample Particles to improve their cellular uptake) to each well, ensuring that each coverslip was dipped properly. After 1 h of incubation, the APTES solution was collected back into the stock solution. The well was rinsed with complete medium twice to remove any traces of APTES. At 90% confluency, cells were harvested from the flask. Then, cells were seeded in 6-well plates at a density of 5,000 to 10,000 cells/well in appropriate medium. The plate was incubated for 24 h at 37°C with 5% CO_2_. Next, the medium was removed, and fresh culture media were added to the well of the plate containing desired concentrations of compound/ formulation and 1 µL per mL of fluorescein isothiocyanate (FITC), and incubated for 24 h. FITC was covalently conjugated to the nanoparticles by reacting with the isothiocyanate group of FITC with the primary amines present on the surface of the nanoparticles. The amine groups were loaded by a surface functionalization with APTES. Reaction was performed in the dark with mild stirring at room temperature for 12–24 h. The nanoparticles labeled with FITC were purified by extensive washing to elute unbound dye from their surface. This covalent coupling results in a stable thiourea bond and ensures predictable fluorescence labeling. After the incubation period, the culture medium was removed, and 1 mL of 3% paraformaldehyde solution was added and incubated for 20 min. Then the plate was rinsed with sterile PBS. 100 µL of 1 µg/mL DAPI (4’,6-diamidino-2-phenylindole) solution was added to the coverslip and incubated for 30 min, and then rinsed with sterile PBS. At last, the coverslip was taken off and placed on a spotless slide [32]. An Inverted Laser Scanning Confocal Microscope (LSM 900, ZEISS) was used to examine the prepared slides. The cellular internalization of CUR-PIP-loaded MNPs was measured through the intrinsic autofluorescence properties of curcumin (green fluorescence; excitation/emission ~420/530 nm). Three groups of images—DAPI, FITC, and Merged—were captured. The software ImageJ (Version 1.54d) was then used to analyze the images and assess their intensity.

#### Statistical analysis

All statistical analyses were conducted using Design-Expert^®^ version 13 software (Stat-Ease Inc., Minneapolis, USA) and GraphPad Prism^®^ version 6 (GraphPad Software Inc., USA). Analysis of variance (ANOVA) was applied to determine the statistical significance of the model and individual terms. A *p*-value of less than 0.05 was considered statistically significant. For cytotoxicity studies, results were expressed as mean ± SEM from triplicate experiments. IC50 was calculated using nonlinear regression analysis with GraphPad Prism software. Differences in cellular uptake were evaluated by image analysis using ImageJ (Version 1.54d).

## Results

### Optimization of CUR-PIP-F68-Fe_3_O_4_ NPs

The MNPs were optimized using a Box-Behnken design, which systematically investigates the influence of three key factors: magnetite concentration (A), concentration of coating solution (B), and sonication time (C) on particle size (R1), zeta potential (R2), and encapsulation efficiency (R3) as shown in Table 2.

**Table 2:** Nanoparticle batches containing factor and response, along with their predicted and observed results.

**Run**	**Independent variable**			**Dependent variable**					
	**A: Concentration of magnetite (%w/v)**	**B: Concentration of coating solution (%w/v)**	**C: Sonication time (minutes)**	**R1: Particle size (nm)**		**R2: Zeta potential (mV)**		**R3: Encapsulation efficiency (%)**	
				**Observed value**	**Predicted value**	**Observed value**	**Predicted value**	**Observed value**	**Predicted value**
**1**	0.25 (0)	1 (0)	45 (0)	240.7 ± 0.31	227.42	–18.3 ± 0.058	–18.94	88.7 ± 0.15	87.63
**2**	0.25 (0)	1 (0)	45 (0)	242.7 ± 1.42	227.42	–19.1 ± 0.153	–18.94	88.5 ± 0.25	87.63
**3**	0.1 (–1)	1.5 (+1)	45 (0)	158.1 ± 1.31	148.48	–25.7 ± 0.458	–25.82	98.85 ± 0.71	98.32
**4**	0.4 (+1)	1.5 (+1)	45 (0)	279.4 ± 0.91	285.26	–17.4 ± 0.200	–16.80	84.43 ± 2.10	85.17
**5**	0.25 (0)	1 (0)	45 (0)	182.4 ± 0.59	227.42	–19.1 ± 0.208	–18.94	88.7 ± 2.54	87.63
**6**	0.4 (+1)	0.5 (–1)	45 (0)	390.6 ± 1.15	400.21	–10.4 ± 0.493	–10.28	60.88 ± 1.04	61.41
**7**	0.25 (0)	1 (0)	45 (0)	235.2 ± 1.16	227.42	–19.1 ± 0.153	–18.94	83.57 ± 1.72	87.63
**8**	0.25 (0)	0.5 (–1)	30 (–1)	355.9 ± 1.86	353.88	–16.6 ± 0.204	–16.56	72.57 ± 0.82	72.44
**9**	0.25 (0)	1 (0)	45 (0)	236.1 ± 2.52	227.42	–19.1 ± 0.153	–18.94	88.7 ± 1.47	87.63
**10**	0.1 (–1)	0.5 (–1)	45 (0)	298.6 ± 0.75	292.73	–21.7 ± 0.100	–22.30	85.8 ± 1.67	85.06
**11**	0.25 (0)	1.5 (+1)	30 (–1)	197.6 ± 0.51	199.34	–21.7 ± 0.200	–22.14	92.1 ± 1.96	91.77
**12**	0.25 (0)	0.5 (–1)	60 (+1)	291.2 ± 1.70	289.46	–18.7 ± 0.153	–18.26	74.11 ± 1.10	74.44
**13**	0.4 (+1)	1 (0)	60 (+1)	309.8 ± 1.08	301.93	–11.9 ± 0.180	–12.46	75.59 ± 0.52	74.73
**14**	0.1 (–1)	1 (0)	60 (+1)	175.6 ± 1.54	183.20	–23.7 ± 0.306	–23.54	94.45 ± 1.08	94.85
**15**	0.25 (0)	1.5 (+1)	60 (+1)	182.8 ± 1.21	184.81	–22.7 ± 0.115	–22.74	92.01 ± 0.29	92.13
**16**	0.4 (+1)	1 (0)	30 (–1)	352.4 ± 1.34	344.80	–11.7 ± 0.252	–11.86	75.67 ± 0.75	75.27
**17**	0.1 (–1)	1 (0)	30 (–1)	211.4 ± 1.67	219.28	–22.4 ± 0.265	–21.84	91.09 ± 1.20	91.95

### Fitting of data to a model

The significant difference between the independent variables (A, B, and C) was evaluated using the ANOVA. The observed response was tailored to different models, i.e., first, second, and quadratic models of Design Expert. The low predicted value of residual error, sum of squares, and high *R*-squared value show that for all responses, the best-fitted model was a quadratic model (“Prob > F” < 0.0001). For dependent variables R1, R2, and R3, the model *F*-value was found to be significant with values 18.75, 96.71, and 47.06, respectively. Furthermore, according to the polynomial equations observed for responses: A, B, C, A^2^, and B^2^, model terms were observed as significant terms for response R1; the model terms A, B, C, AB, A^2^, and B^2^ were found to be significant terms for response R2. For response R3, the significant model terms were identified as A, B, AB, and B^2^. For all responses, R1, R2, and R3, the lack of fit *F*-value was found to be 0.26, 4.75, and 0.24, respectively. These observations pointed towards the statistic that the lack of fit was insignificant and related to pure error. For responses R1, R2, and R3, the *R*-squared values were found to be 0.960, 0.992, and 0.984, respectively. The adequate precision values were 15.65, 35.11, and 25.79 for responses R1, R2, and R3, respectively. A value of more than four was desirable for adequate precision. Table 3 [mean ± SD (*n* = 3)] gives the statistical summary for all responses.

**Table 3:** Statistics summary of all responses (R1, R2, and R3).

**Parameters**	**Df**	**Sum of squares**	**Mean**	***F*-value**	***P*-value**	***R* ^2^**	**Adjusted *R*^2^**	**Predicted *R*^2^**	**S.D.**	**CV%**	**Adequate precision**
**Particle size**											
Model	9	74,261.54	8,251.28	18.75	0.0004	0.9602	0.9090	0.8431	20.98	8.22	15.64
Residual	7	3,079.74	439.96								
Lack of fit	3	507.23	169.08	0.2629	0.8494						
Pure error	4	2,572.51	643.13								
Cor total	16	77,341.27									
**Zeta potential**											
Model	9	290.27	32.25	96.71	< 0.0001	0.9920	0.9818	0.8976	0.5775	3.07	35.11
Residual	7	2.33	0.3335								
Lack of fit	3	1.82	0.6075	4.75	0.0833						
Pure error	4	0.5120	0.1280								
Cor total	16	292.60									
**Encapsulation efficiency**											
Model	9	1,475.12	163.90	47.06	< 0.0001	0.9837	0.9628	0.9389	1.87	2.21	25.79
Residual	7	24.38	3.48								
Lack of fit	3	3.71	1.24	0.2389	0.8654						
Pure error	4	20.68	5.17								
Cor total	16	1,499.50									

#### Response 1 (R1): Effect of independent variables on particle size

In nanoparticle drug delivery systems, particle size is the utmost criterion. The polynomial equation for response R1 is given below:

R1 particle size=+227.42+61.06A−64.80B−19.74C+7.33AB+1.70AC+12.48BC+29.84A2+24.42B2+5.04C2

**Figure 2:**
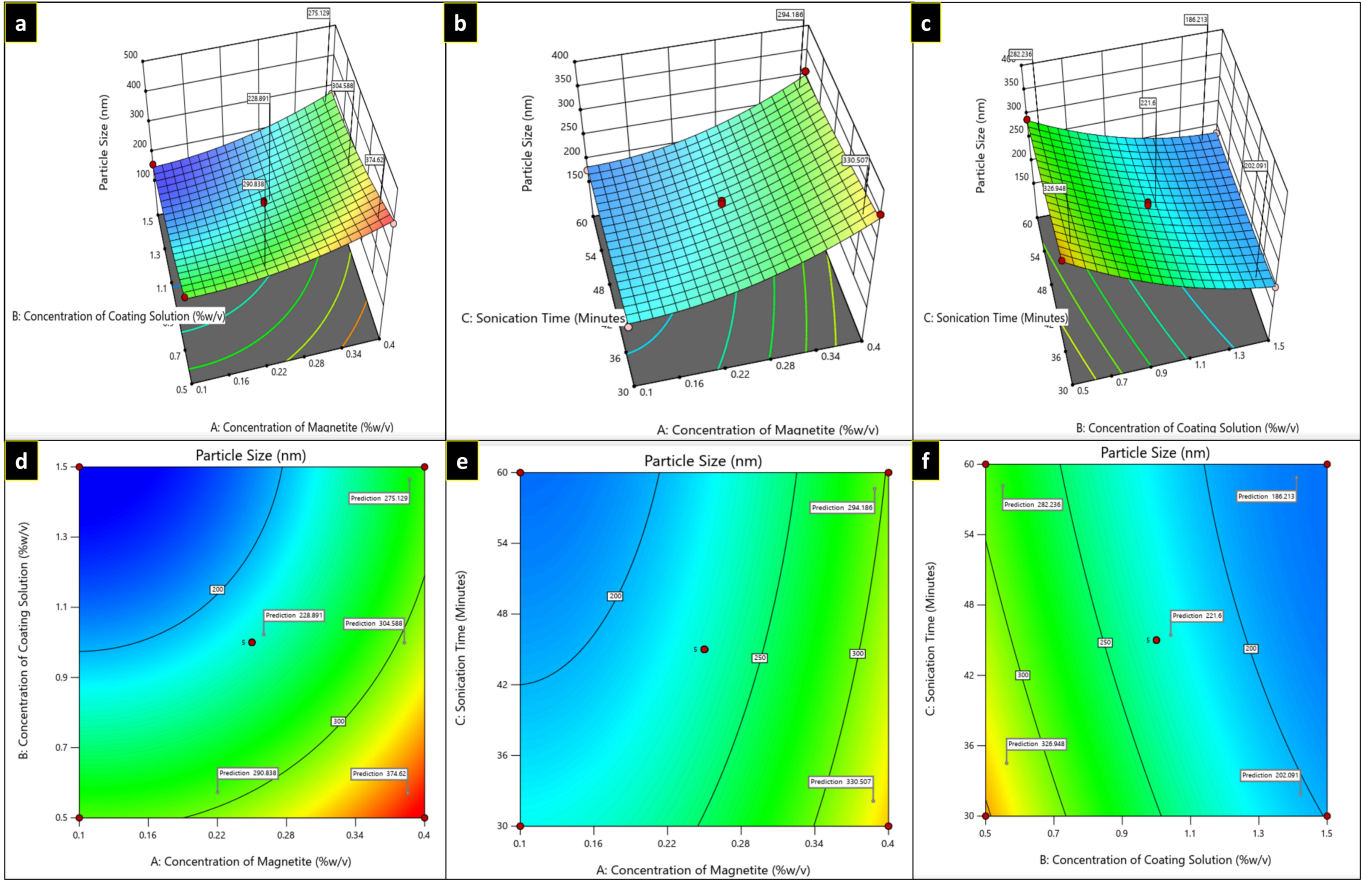
Three-dimensional plots (a–c) and contour plots (d–f) showing the effect of independent variables on particle size.

**Figure 3:**
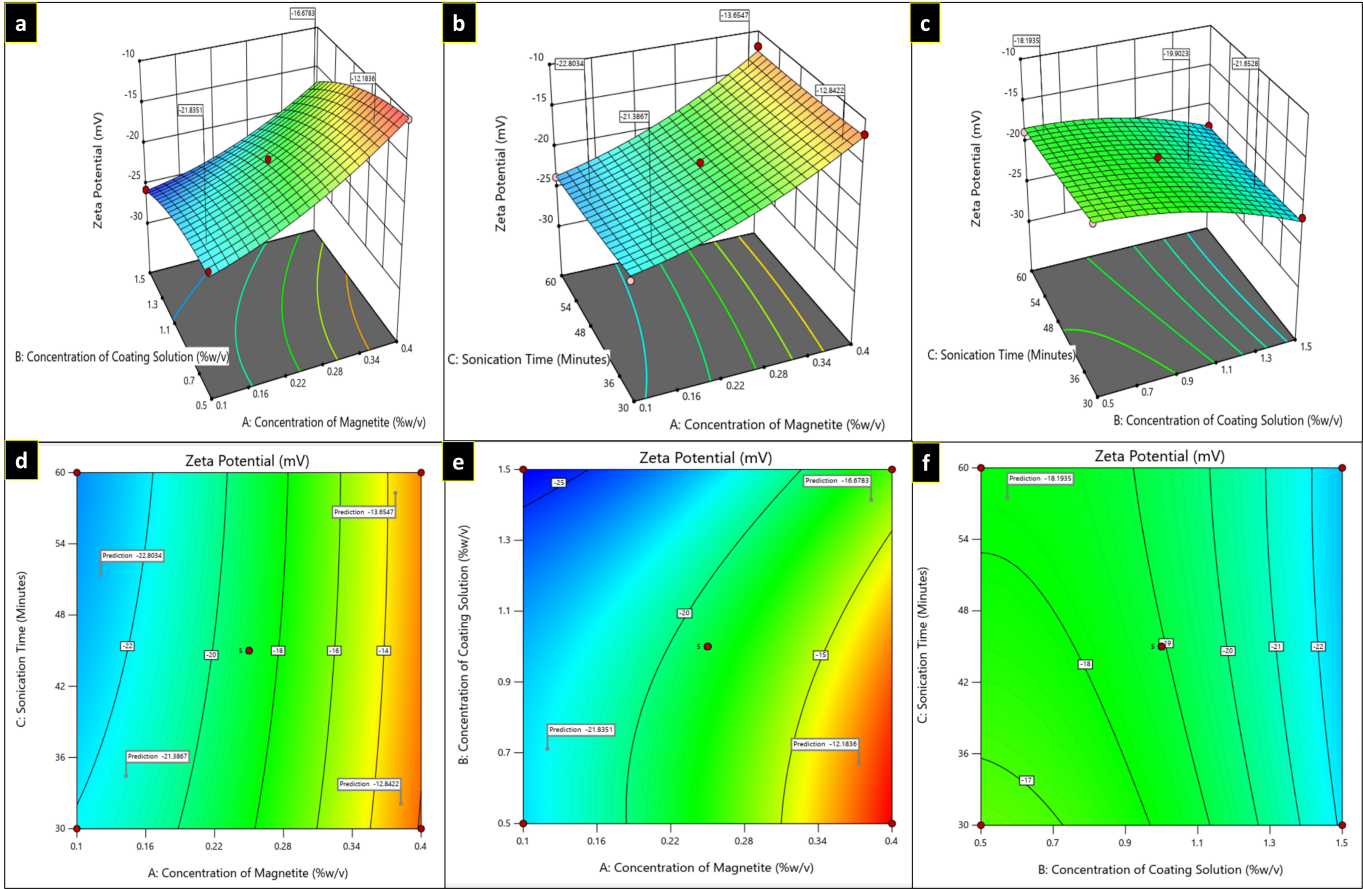
Three-dimensional plots (a–c) and contour plots (d–f) showing the effect of independent variables on zeta potential.

**Figure 4:**
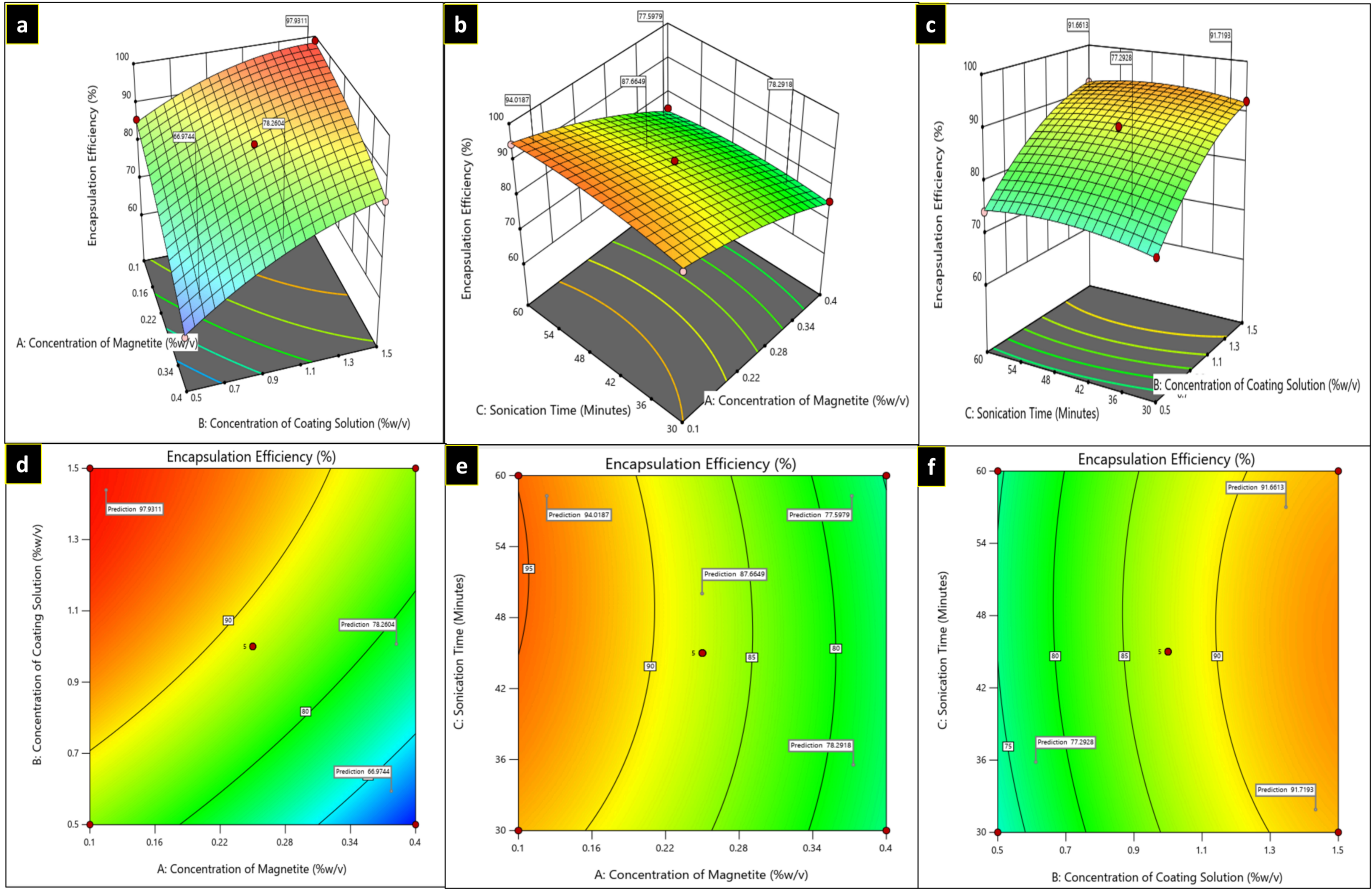
Three-dimensional plots (a–c) and contour plots (d–f) showing the effect of independent variables on encapsulation efficiency.

Three-dimensional (3D) plots and contour plots help to explicate the interactions between the formulation variables and the dependent variable (Figures 2, 3, and 4).

For R1 response, in the equation, a positive value of A represents that particle size increases as the concentration of magnetite increases, while with B and C, a negative coefficient was observed, representing a decrease in particle size as the concentration of coating solution and sonication time increased, respectively, as illustrated in Figure 2. Table 2 shows the average particle size of several formulations of MNPs. The greater particle size (390.6 ± 1.15 nm) was observed at low coating solution concentrations (0.5% w/v) surrounding the magnetite core. However, with the increase in concentrations of independent variable B (1.5% w/v) and C, sonication time, particle size was found to be decreased (158.7 ± 1.31 nm).

#### Response 2 (R2): Effect of independent variables on zeta potential

The polynomial equation for response 2 is given below:

R2 zeta potential=−19.73417+10.25A−5.265B−0.183C−10.00AB+0.12222AC+0.0366BC+58.66A2−4.72B2+0.00086C2

**Figure 5:**
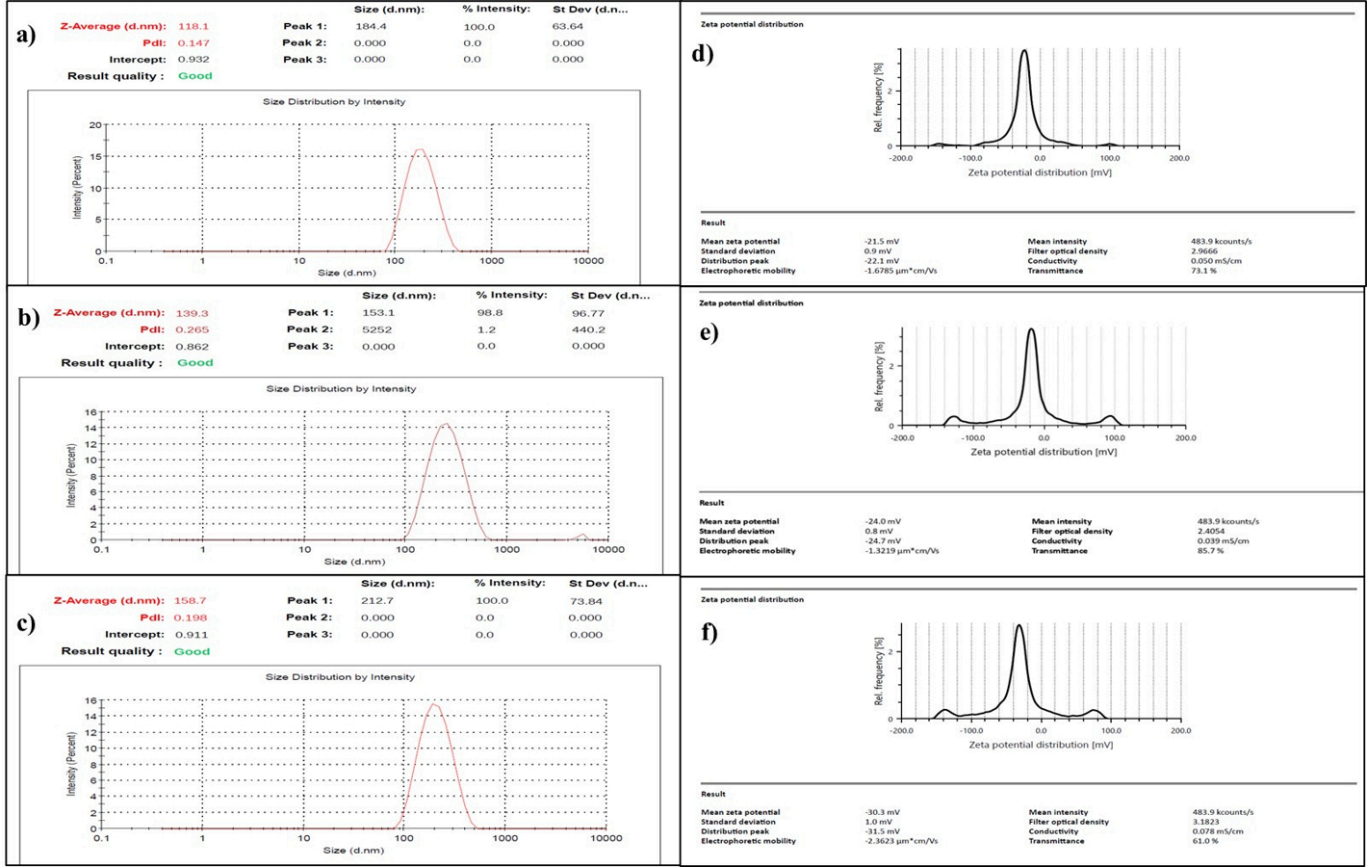
**Particle size (a–c) and Zeta potential (d–f) of bare MNPs, MNPs with coating, and CUR-PIP-F68-Fe_3_O_4_ NPs, respectively.** MNPs: magnetic nanoparticles.

For R2 response, in the equation, a positive value of A (magnetite concentration) signifies that the zeta potential declines (to become less negative) as the magnetite concentration increases suggesting a possible reduction in colloidal stability, while with B (concentration of coating solution) and C (sonication time), a negative coefficient was observed, imply that increasing either variable result in a more negative zeta potential, thereby enhancing the electrostatic stabilization of the nanoparticle dispersion. It has been observed that with an increase in coating solution, zeta potential increases to the negative side (–21.3 mV to –30.3 mV as depicted in Figure 5). From the plots (Figure 3), it was depicted that zeta potential decreases to the positive side (to become less negative) as the concentration of the independent variable A (magnetite) increases.

#### Response 3 (R3): Effect of independent variables (A, B & C) on encapsulation efficiency.

The polynomial equation for response 3 is given below:

R3 encapsulation efficiency=+51.825−38.69A+38.79B+0.834C+35.000AB−0.382AC−0.054BC−80.92A2−13.29B2−0.007C2

For the R3 response, in the equation, the negative value of A (concentration of magnetite) indicates that encapsulation efficiency decreases as the magnetite concentration increases. The positive coefficient of B and C shows that the encapsulation efficiency increased as the concentration of coating solution (B) and sonication time (C) increased. The minimum and maximum encapsulation efficiency values for curcumin-piperine loaded Poloxamer F-68 coated magnetic nanoparticles (CUR-PIP-F68-Fe_3_O_4_ NPs) observed were 60.88 ± 1.04% for F6 and 98.85 ± 0.71% for F3, respectively, as depicted in Table 2. The 3D response surface plots and contour plots for response 3 are given in Figure 4.

#### Optimization of drug-loaded formulation

The point prediction method of Design Expert software was applied to optimize the developed formulation. Five optimum checkpoint formulations (Table 4) were prepared based on criteria accomplishing minimum particle size with maximum encapsulation efficiency and zeta potential. The desirability found for the optimised formulation was 0.998. Predicted formulations were formulated in the laboratory, and further characterization, such as particle size, zeta potential of the particles, and encapsulation efficiency, was measured. It is clear from the results shown in Table 4 that the formulation with 0.1% w/v of magnetite, 1.5% w/v of coating solution, and a sonication time of 52.82 min shows good agreement with the predicted values. For dependent variables R1, R2, and R3, the optimal values were determined to be 158.7 ± 0.057 nm, –30.3 ± 0.100 mV, and 98.85 ± 0.066%, respectively. For further analysis, the selected optimal formulation was employed.

**Table 4:** Composition of checkpoint formulation.

**Formulation composition**	**Response variable**	**Experiment value (*n* = 3)**	**Predicted value**	**%Predicted error**
**A:B:C**				
0.1:1.5:52.82	R1	158.7 ± 0.057	158.4	–0.1894
	R2	–30.3 ± 0.100	–25.99	–16.583
	R3	98.85 ± 0.066	98.49	–0.365
0.138:1.5:60	R1	181.1 ± 1.738	178.3	–1.570
	R2	–26.62 ± 0.011	–25.67	–3.700
	R3	96.01 ± 0.073	96.93	0.949
0.169:1.5:33.93	R1	189.4 ± 0.265	190.7	0.682
	R2	–23.7 ± 0.057	–24.25	2.268
	R3	94.20 ± 0.251	95.23	1.081
0.105:1.386:45.74	R1	195.90 ± 0.351	198.64	1.379
	R2	–21.5 ± 0.116	–22.70	5.286
	R3	93.75 ± 0.189	94.30	0.583
0.260:1.5:30	R1	205.60 ± 1.011	204.13	–0.720
	R2	–18.9 ± 0.076	–21.85	13.501
	R3	87.89 ± 0.030	91.38	3.819

#### Particle size determination

Particle size of bare MNPs (magnetic core of iron oxide; Fe_3_O_4_), poloxamer F-68 coated MNPs, and CUR-PIP-F68-Fe_3_O_4_ MNPs was measured using a particle size analyser (diffraction laser spectroscopy). It has been reported in a number of studies that the size obtained by the DLS technique is affected by the thickness of the electrical double layer and adsorption of the material on the surface of nanoparticles [33, 34]. The average hydrodynamic diameters for bare MNPs, MNPs with polymer coating, and drug-loaded MNPs were found to be 118.1 ± 0.12 nm, 139.3 ± 1.33 nm, and 158.7 ± 0.057 nm, respectively, as shown in Figure 5a–c.

#### Zeta potential

Magnetite possesses the limitations of aggregation. To prevent aggregation or to provide colloidal stability, magnetite was coated with Pluronic F-68. Further, the colloidal stability of the particles was measured by zeta potential determination. The zeta potential results for bare MNPs, MNPs with polymer coating, and CUR-PIP-F68-Fe_3_O_4_ NPs were observed to be –21.5 ± 0.115 mV, Figure 5d, –24.0 ± 0.153 mV, Figure 5e, and –30.3 ± 0.252 mV, Figure 5f, respectively.

#### Encapsulation efficiency

The optimized MNPs of CUR-PIP-F68-Fe_3_O_4_ NPs exhibited a high drug encapsulation efficiency of 98.85 ± 0.066%. This impressive level of entrapment indicates that most of the drug was successfully incorporated into the nanoparticles, which minimizes drug loss and enhances the MNPs’ therapeutic potential. Such high encapsulation efficiency is crucial for maximizing drug delivery effectiveness, reducing required dosages, and minimizing side effects [35].

#### Scanning electron microscopy-energy dispersive spectroscopy (SEM-EDS)

**Figure 6:**
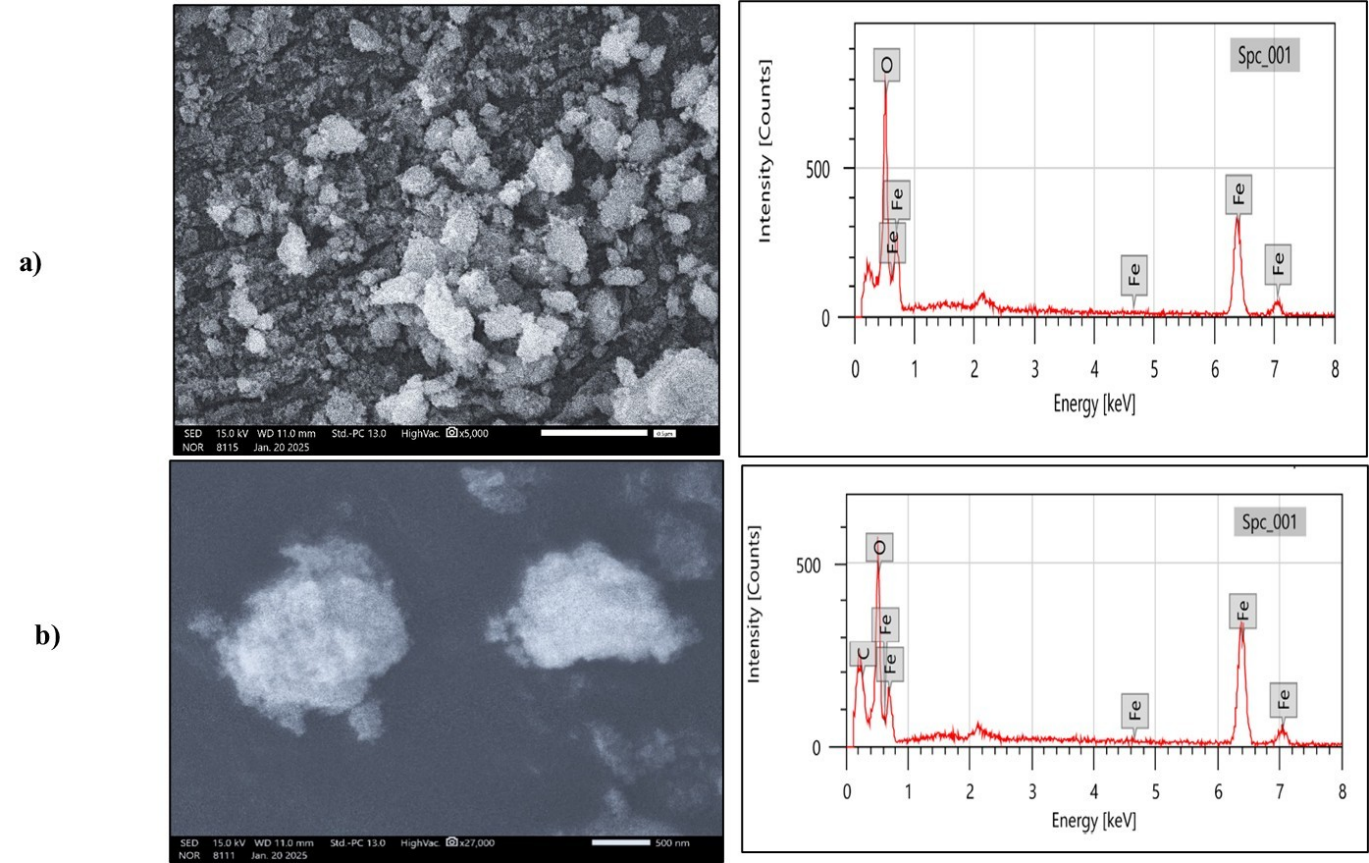
SEM images and EDX graph of a) bare MNPs, b) optimised CUR-PIP-F68-Fe_3_O_4_ NPs.

SEM-EDS analysis for Bare MNPs and CUR-PIP loaded Poloxamer F-68 Coated MNPs was performed, and results are depicted in Figure 6 and Table 5. SEM images showed that the synthesized nanoparticles are spherical but appear as clusters; aggregation can be observed in the case of Bare MNPs, whereas the aggregation has been minimized in the case of coated MNPs. The increased mass percentage of carbon (13.50 ± 0.30%) in the curcumin-piperine-loaded, poloxamer F-68-coated MNPs, compared to 3.50 ± 0.11% in bare Fe_3_O_4_, confirms successful surface modification and loading of curcumin and piperine.

**Table 5:** EDX composition for bare MNPs and CUR-PIP-F68-Fe_3_O_4_ MNPs.

**Element**	**Mass %**	**Atom %**
EDX bare MNPs		
C	3.50 ± 0.11	6.39 ± 0.05
O	19.61 ± 0.32	44.81 ± 0.72
Fe	76.89 ± 1.21	48.80 ± 0.77
Total	100.00	100.00
EDX F-68 coated MNPs		
C	13.50 ± 0.30	26.38 ± 0.38
O	7.69 ± 0.29	23.37 ± 0.65
Fe	78.81 ± 1.23	50.25 ± 0.78
Total	100.00	100.00

#### TEM

**Figure 7:**
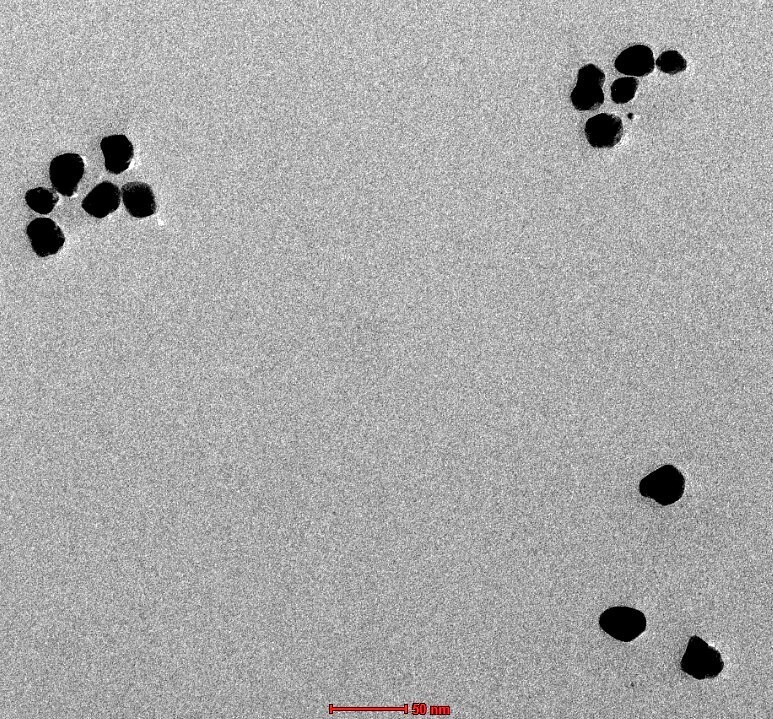
Transmission electron microscopy images of CUR-PIP-F68-Fe_3_O_4_ nanoparticles.

TEM depicts morphological characterization by visualizing clear images of the observed sample [36]. TEM results of the prepared CUR-PIP-F68-Fe_3_O_4_ MNPs are shown in Figure 7. It can be seen from the picture that the prepared MNPs are spherical.

#### VSM analysis

**Figure 8:**
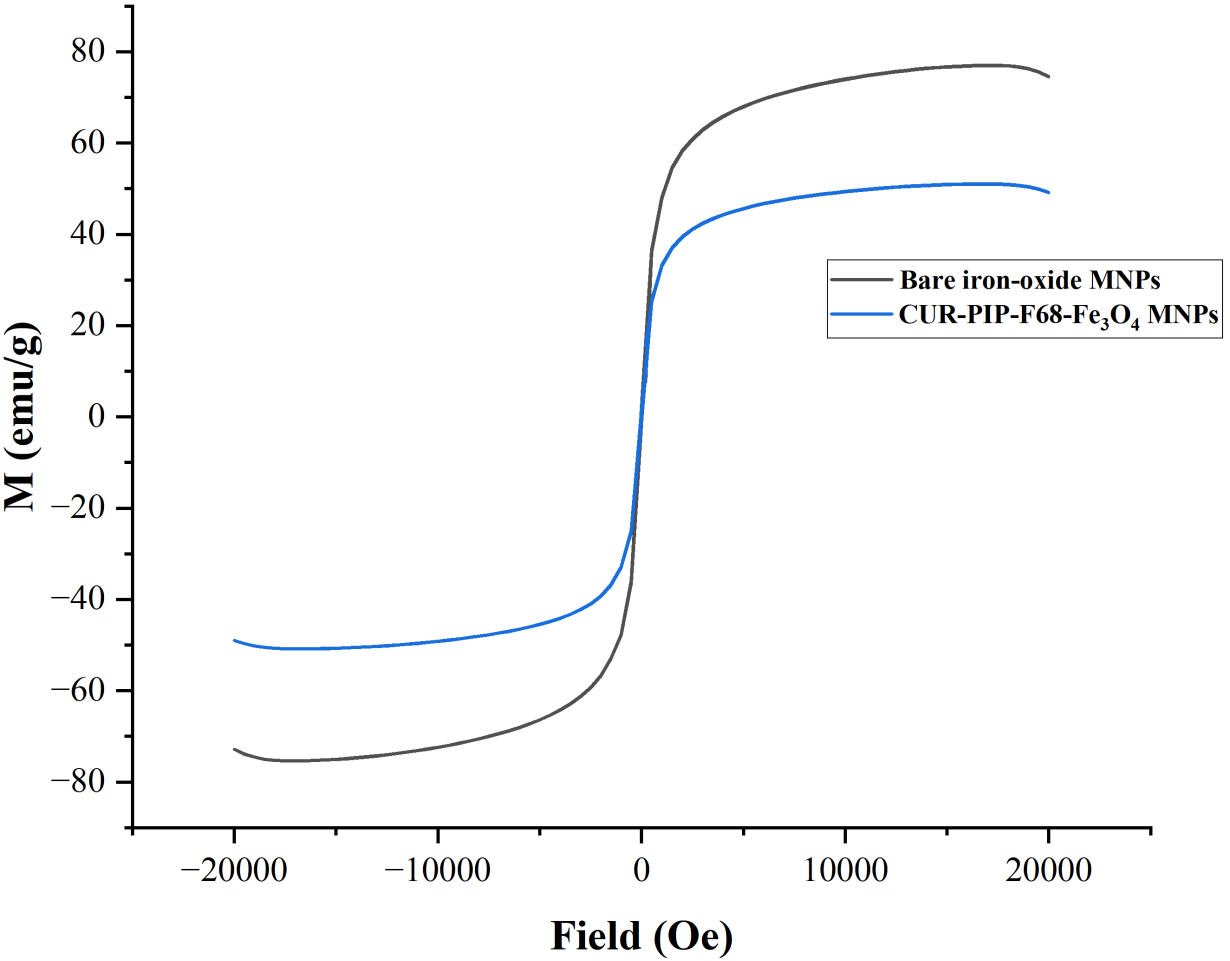
Magnetization curve of bare MNPs and CUR-PIP-Poloxamer F-68 loaded Fe_3_O_4_ nanoparticles.

The magnetic characteristics of bare MNPs and CUR-PIP-F68-Fe_3_O_4_ nanoparticles were determined by measuring magnetization as a function of field. Figure 8 shows the acquired data [saturation magnetization (Ms)]. The saturated magnetization of CUR-PIP-F68-Fe_3_O_4_ nanoparticles was found to be 50.7 emu/g, compared to bare MNPs (Fe_3_O_4_ nanoparticles), which possess the Ms of 75.6 emu/g.

### In vitro cytotoxicity assay

**Figure 9:**
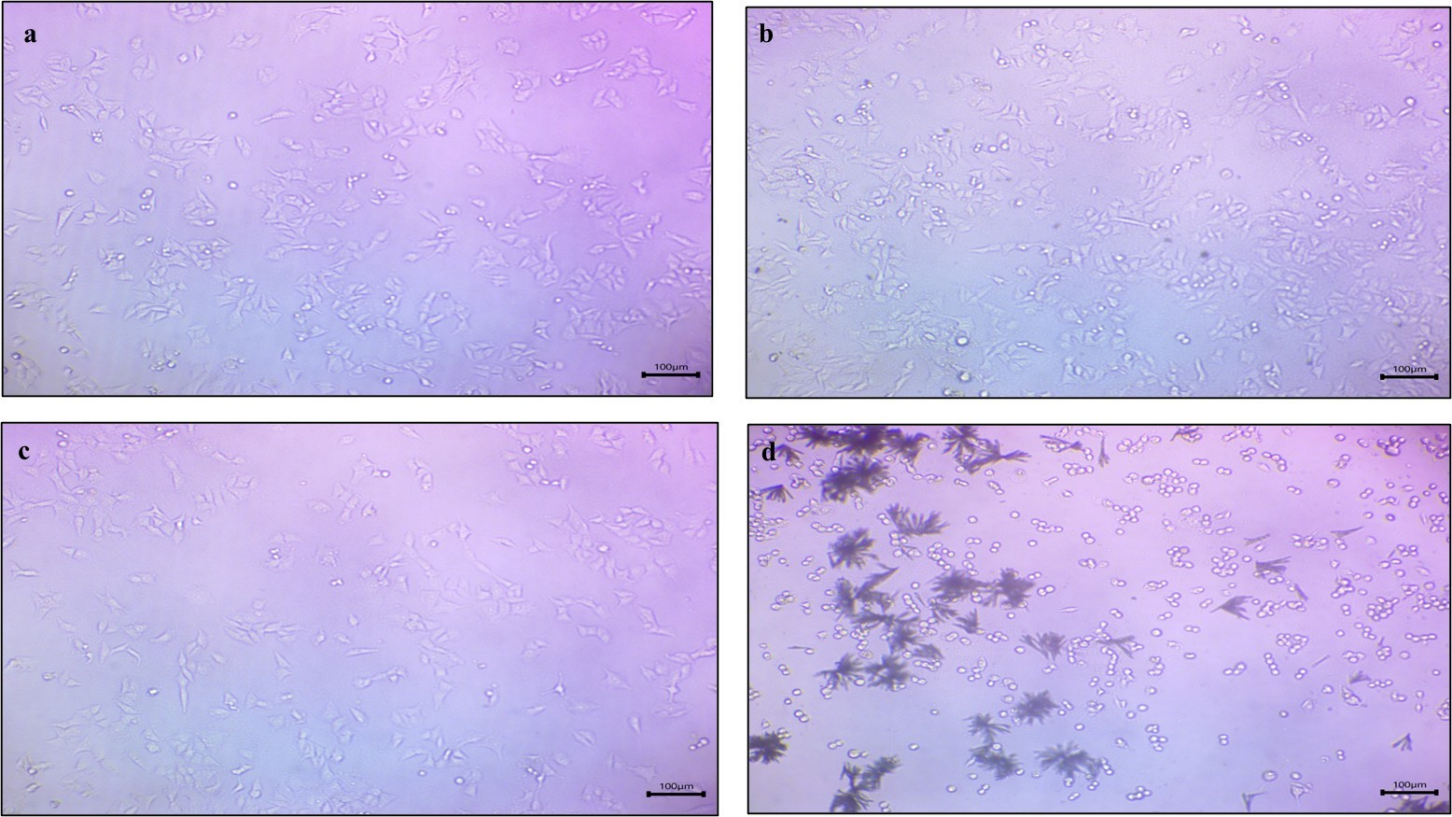
Microscopy images of a) free curcumin and piperine drug, b) curcumin MNPs, c) piperine MNPs, and d) CUR-PIP-F68-Fe_3_O_4_ NPs (scale: 100×).

The viability of Colorectal HCT-116 cancer cells was measured using an MTT assay on treating cells with free curcumin and piperine, curcumin MNPs, piperine MNPs, and a combination of curcumin and piperine MNPs at various concentrations (1 to 200 mcg/mL) for 24 h. Brightfield microscopic images of all samples reveal the morphological characteristics of the cells throughout the experiment when treated with different concentrations. It has been revealed from the pictures (Figure 9) that no changes in cells were observed in control and piperine MNPs throughout the experiment, whereas round-shaped cells were observed in the curcumin MNPs and the sample with a combination of curcumin and piperine MNPs after treatment for 24 h.

**Figure 10:**
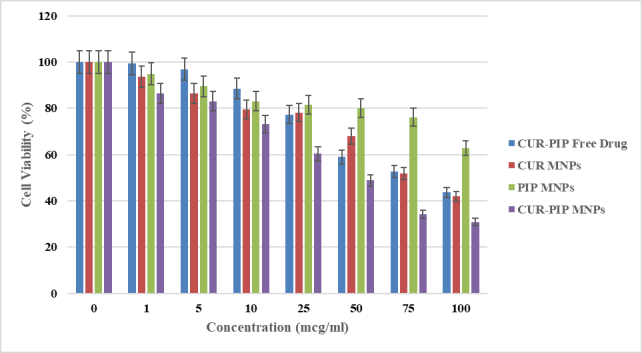
% cell viability graph as a function of increasing concentration on the HCT-116 cell line.

Results shown in Figure 10 depict the % viability data of various samples with an increase in concentration against the HCT-116 cancer cell line for 24 h. It has been observed that the cell viability of CUR-PIP-F68-Fe_3_O_4_ nanoparticles was significantly decreased to 30.86% as compared to individual MNPs of curcumin (41.91%) and piperine (62.68%). Using the % viability data, further IC50 values were estimated, and values were found to be 87.47 ± 0.28, 79.68 ± 0.41, 115.84 ± 0.25, and 50.24 ± 0.57 mcg/mL for free curcumin and piperine drug, curcumin MNPs, piperine MNPs, and a combination of curcumin and piperine MNPs, respectively.

### Cellular uptake studies

**Figure 11:**
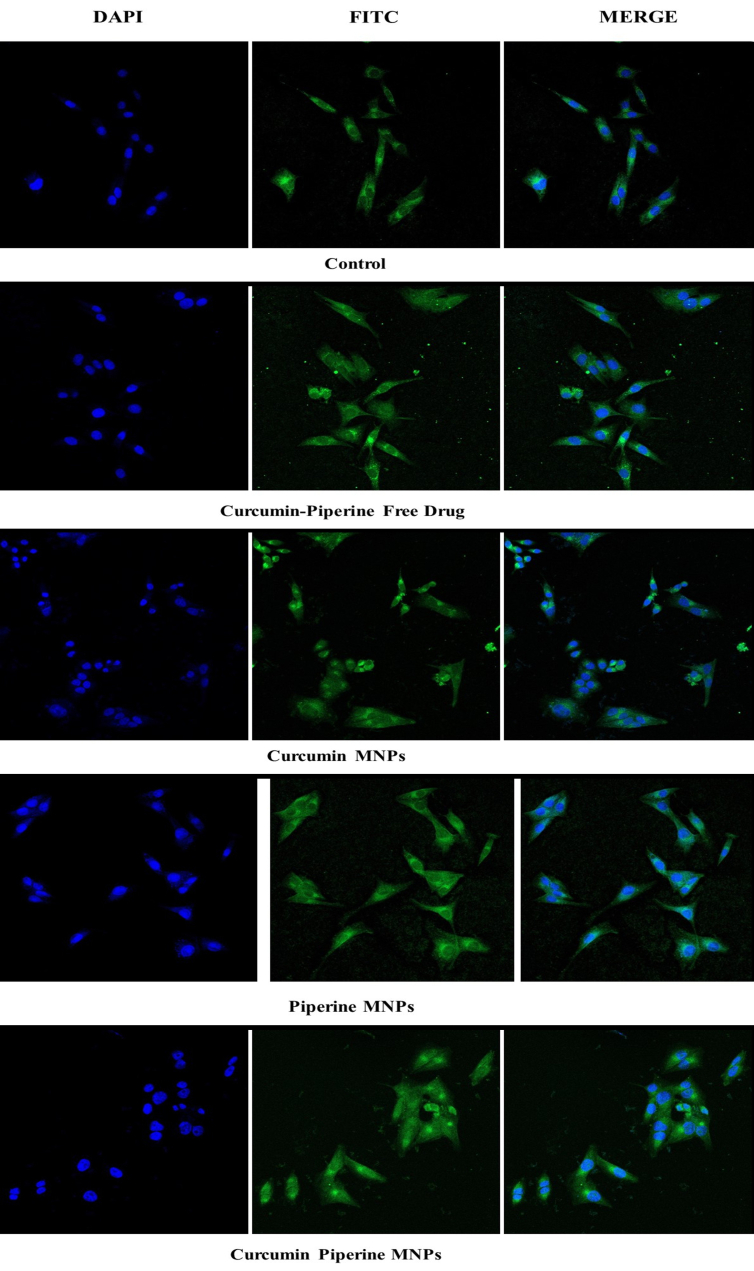
Confocal microscopy images depicting the % fluorescence intensity of I) control, II) free curcumin and piperine drug, III) Curcumin MNPs, IV) piperine MNPs, and V) CUR-PIP-F68-Fe_3_O_4_ MNPs by HCT-116 cells (scale: 40×, magnification: 10 µm).

The uptake of MNPs loaded with curcumin-piperine into HCT-116 cells was observed using confocal microscopy, as depicted in Figure 11. The results confirmed that the highest amount of cellular uptake was observed in curcumin-piperine MNPs, which appears to be 2.49-fold higher than that of control cells; the lowest uptake was observed with fluorescence intensity about 45.95% in piperine MNPs compared to control (53.96%), showing higher potential of combination therapy.

## Discussion

Numerous modern drug delivery systems are specifically designed to optimize the delivery of a specific drug form by minimizing side effects, eliciting bioavailability, promoting and encouraging the accumulation of the pharmaceutical drug at the required biozone (site), minimizing degradation or loss of the drug, and addressing other difficult problems related to therapeutic delivery targeting or the physical stability of the drug [37, 38]. However, developing a delivery method with minimized hazardous side effects and optimal therapeutic action, particularly in vivo, is difficult.

This present study aimed to develop active phytoconstituents loaded MNPs to reduce side effects and achieve targeted drug delivery. To prepare the MNPs (also known as Fe_3_O_4_ NPs or magnetite), the co-precipitation approach was adopted [39]. Selecting a suitable polymer for preparing iron oxide MNPs is crucial for achieving stability, functionality, and desired properties. As per literature reports, biocompatibility, aqueous solubility, surface functionalization, colloidal stability, and targeted delivery are the major considerations when selecting a polymer [40, 41]. Poloxamer F-68 was selected for the study based on the results obtained for the desired particle size.

The nanoparticles were further optimized using a Box-Behnken design, which systematically investigates the influence of three key factors: magnetite concentration (A), concentration of coating solution (B), and sonication time (C). This design is beneficial as it allows for the evaluation of both the individual effects of each factor and their interactions, offering a comprehensive understanding of their contributions to the performance of a nanocarrier. Particle size (R1) and encapsulation efficiency (R3) measure the proportion of the active ingredient successfully incorporated into the carriers, reflecting the formulation’s effectiveness [42].

Particle size affects the stability, bioavailability, and release profile of the drug. Zeta potential represents the surface charge of the nanoparticles, influencing their stability and interaction with biological membranes. This approach aimed to identify optimal levels for each factor and uncover potential synergies or antagonisms between them. These interactions play a crucial role in shaping the characteristics and performance of the nanosystem [43]. The decrease in particle size with increasing coating solution concentration from 390.6 ± 1.15 nm to 158.7 ± 1.31 nm suggests that both factors contribute to improved colloidal stability and reduced agglomeration, likely due to changes in surface functionalization. Another reason is that with probe sonication, the cavitation phenomenon occurs when ultrasonic waves are applied to a prepared dispersion of solid particles and solvent. Microbubbles are produced and then implosively collapse in the liquid throughout this process. Smaller droplets in the phase break up and disperse due to the continuous sonication, creating strong turbulence during the collapse of the microbubbles and the hot spots created by this transient cavitation process that diminish particle size [44]. The dispersed media can interact with the shear force produced by the acoustic cavitation process for a longer period when the sonication time is increased [45]. A low PDI value indicates a narrow size distribution and high uniformity, which are desirable characteristics for consistent performance in subsequent applications. In the case of the effect of independent variables on zeta potential, it has been observed that with an increase in coating solution, zeta potential increases to the negative side (–21.3 mV to –30.3 mV). Factors influencing zeta potential include solution pH, ionic strength, and ionic species. The addition of NPs would alter the initial solution pH due to the protonation or deprotonation of their surface groups. In one of the studies reported by Suttiponparnit et al. [46], it was discovered that the solution pH declined from 5.72 to 5.08 when the particle concentration increased from 15 to 500 mg L^–1^. They concluded that the drop in solution pH produces an increase in the zeta potential of oxide particles at high particle concentrations, which often flips to a more positive value as pH decreases.

SEM images showed that the synthesized nanoparticles are spherical, but aggregation can be observed in the case of Bare MNPs, that is attributed to the magnetic interactions between Fe_3_O_4_ MNPs, whereas the aggregation has been minimized in the case of coated MNPs due to a change in surface functionalization. The EDX pattern indicates the existence of Fe, O, and C elements. The weight % of C (13.50 ± 0.30), O (7.69 ± 0.29), and Fe (78.81 ± 1.23) in CUR-PIP-F68-Fe_3_O_4_ NPs indicated that surface modification by coating of poloxamer F-68 on the surface of MNPs [47]. Major enhancement in carbon content as compared to bare MNPs (3.50 ± 0.11%) could be attributed to poloxamer F-68 coating and the loaded bioactive compounds (curcumin and piperine) as carbon-rich organic entities.

TEM provides additional morphological images of the synthesized CUR-PIP-Fe_3_O_4_ nanoparticles as illustrated in Figure 7. The diameter or scale of the particles detected in TEM is less than that found by the DLS method because of the physical state of the samples under visualization. Following coating with Poloxamer F-68, the prepared Fe_3_O_4_ nanoparticles demonstrated consistent spherical morphologies and verified the increase in particle size (observed by DLS method, Figure 5a–c. Consequently, this suggests that the polymer adsorption on the surface of nanoparticles has changed the functionality, hence, minimised the aggregation [48]. Muniyappan et al. (2021) [49] developed gold particles using *Curcuma pseudomontana* isolated curcumin. According to the results published, formulated nanoparticles were spherical in shape, evenly distributed, and had an average particle size of 39 nm. TEM measurements and size distribution analyses of the synthesized gold nanoparticles showed that the particles are spherical with an average diameter of 20 nm and within 10% of the size distribution.

Figure 8 shows the acquired data Ms. The saturated magnetization of CUR-PIP poloxamer F-68 coated nanoparticles was found to be 50.7 emu/g, compared to bare MNPs, which possess the Ms of 75.6 emu/g. Nosrati et al. [50] synthesized bovine serum albumin-coated curcumin iron oxide nanoparticles through desolvation and co-precipitation. During this work, the lessening of Ms for coated MNPs (22 emu/g) over bare ironoxide nanoparticles (70 emu/g) can be elucidated by the coating layer on the surface of Fe_3_O_4_ nanoparticles.

An MTT assay was used to assess the vitality of colon cancer cells (HCT-116) after they were exposed to free curcumin and piperine, curcumin MNPs, piperine MNPs, and a combination of curcumin and piperine MNPs at various concentrations (1 to 200 mcg/mL) for 24 h. Brightfield microscopic images of all samples reveal the morphological characteristics of the cells throughout the experiment when treated with different concentrations. It has been revealed from the pictures (Figure 9) that no changes in cells were observed in control and piperine MNPs throughout the experiment, whereas round-shaped cells were observed in the curcumin MNPs and the sample with a combination of curcumin and piperine MNPs after treatment for 24 h. Curcumin MNP treatments caused cells to change into rounder cell morphologies, which is explained by the loss of integrity of HCT-116 cells and detachment due to apoptosis [51]. Apoptosis was evident from the observed morphological alterations, which included aggregation, spherical form, and shrinkage [52, 53]. Additionally, an increase in the number of cells with lost integrity was noted in formulation with a combination of curcumin and piperine MNPs. The rise in the quantity of cells that had lost their integrity was also ascribed to the biological impact of piperine [54].

IC50 values were estimated and were found to be 87.47 ± 0.28, 79.68 ± 0.41, 115.84 ± 0.25, and 50.24 ± 0.57 mcg/mL for free curcumin and piperine drug, Curcumin MNPs, Piperine MNPs, and a combination of curcumin and piperine MNPs, respectively, based on the 24-hour cell viability data (Figure 10). The results demonstrate that MNPs encapsulated with CUR-PIP have a greater cytotoxicity impact than individual curcumin. As demonstrated in multiple papers [55, 56], this suggests that the nanoprecipitation method can increase the bioavailability of CUR and/or PIP in cancer cells, hence intensifying its lethal action. These findings are consistent with those of Abolhassani et al. [57] about the self-assembly approach used to generate CUR-PIP-loaded human serum albumin (HSA) nanoparticles. Their results show that CUR-PIP-HSA nanoparticles are highly cytotoxic, which may be explained by Piperine dual functions as a bio-enhancer and anti-cancer agent. Compared to CUR-HSA nanoparticles, CUR-PIP-HSA nanoparticles exhibited noticeably greater cell inhibition rates. Compared to PIP-HSA nanoparticles, CUR-PIP-HSA nanoparticles had a higher cytotoxic effect on MCF-7 cells.

To better understand the synergistic effect of the two phytoconstituents and assess the potential of MNPs to facilitate the target delivery of these compounds to the specific cells, the biological efficacies of curcumin and piperine MNPs were observed in vitro on an HCT-116 colon cell line model, both individually and in combination. Sample molecules of interest are labeled with FITC in cellular uptake experiments so that their internalization into cells may be monitored [58]. A fluorescent dye called DAPI is frequently used to identify DNA in cells. It is generally employed to investigate the cellular absorption of different compounds and to see the cell nucleus [59, 60]. When DAPI is excited by UV light, it attaches to the minor groove of double-stranded DNA and fluoresces blue. As a counterstain, DAPI is used to identify the cell nucleus and investigate how cells absorb chemicals. The quantity of chemicals absorbed by cells can be measured using the fluorescence of DAPI.

The autofluorescence characteristics of curcumin enable tracking the cell absorption of curcumin MNPs while investigating the uptake of curcumin magnetic nanoparticles (CUR MNPs) independently [61]. Both with and without piperine MNPs, the internalization of CUR MNPs was seen in Figure 11. A higher uptake was ascribed to the higher fluorescence intensity of CUR MNPs in the presence of PIP MNPs. These results are consistent with other research showing that piperine addition greatly increases the amount of curcumin absorption. Similarly, Lund and Pantuso showed that curcumin’s permeability across intact Caco-2 monolayers increased by 229% upon the addition of piperine [62].

### Conclusion

The present study successfully synthesized and optimized curcumin and piperine-loaded MNPs (CUR-PIP-F68-Fe_3_O_4_ MNPs) using the co-precipitation method, with optimization facilitated by the Box-Behnken design. The optimized nanoparticles demonstrated favorable physicochemical properties, morphological and elemental analyses confirmed the successful formulation of the nanoparticles, and magnetic studies revealed substantial magnetic saturation values. The in vitro cytotoxicity results, supported by MTT assays and confocal microscopy, confirmed enhanced cellular uptake and a synergistic anticancer effect of the CUR-PIP-F68-Fe_3_O_4_ MNPs against the HCT-116 colon cancer cell line. These findings suggest that the co-delivery of curcumin and piperine via MNPs would significantly improve the therapeutic efficacy. In this work, MNPs were used mainly for convenient separation and purification based on their excellent magnetic response. Nevertheless, the magnetic core, normally Fe_3_O_4_, also endows the nanoparticles with multifunctionality, which is of great interest for advanced biomedical applications. Importantly, MNPs have the potential to target specific lesion areas when subjected to an external magnetic field (magnetic targeted drug delivery). Additionally, iron oxide NPs have been significantly investigated and were even approved as T2-weighted contrast agents in MRI, allowing for non-invasive diagnostics. Although this study has not experimentally evaluated the magnetic targeting or imaging capabilities of the synthesized MNPs, the structural and magnetic properties of the MNPs designed are theoretically promising for such applications. Future studies will address assessing the in vitro and in vivo magnetic targeting performance, MRI contrast enhancement, as well as comparative assays using normal human cells to assess the therapeutic selectivity in order to optimize the therapeutic and diagnostic features of these multifunctional nanocarriers.

## Abbreviations

APTES: 3-aminopropyltriethoxysilane
CUR MNPs: curcumin magnetic nanoparticles
CUR-PIP-F68-Fe_3_O_4_ NPs: curcumin-piperine loaded Poloxamer F-68 coated magnetic nanoparticles
DAPI: 4’,6-diamidino-2-phenylindole
FITC: fluorescein isothiocyanate
HSA: human serum albumin
IC50: half-maximal inhibitory concentration
MNPs: magnetic nanoparticles
Ms: saturation magnetization
PDI: polydispersity index
SEM-EDS: scanning electron microscopy-energy dispersive spectroscopy
SEM-EDX: scanning electron microscopy-energy dispersive X-ray
TEM: transmission electron microscopy
VSM: vibrating simulating magnetometer

## Declarations

### Availability of data and materials

All data generated/analysed through experimentation and interpreted is included in the research article.

### Copyright

© The Author(s) 2025.
